# The mitochondrial theory of sleep: an integrative framework for understanding sleep-wake regulation

**DOI:** 10.3389/fpsyt.2026.1922556

**Published:** 2026-09-07

**Authors:** Seithikurippu R. Pandi-Perumal, Sayan Paul, Konda Mani Saravanan, Arehally M. Mahalakshmi, Saravana Babu Chidambaram, Ganesh Pandian Namasivayam

**Affiliations:** 1 Central Research Facility (CRF), Dr D.Y Patil Medical College Hospital and Research Centre, Dr D.Y Patil Vidyapeeth, Pune, India; 2 Centre for Research and Development, Chandigarh University, Mohali, Punjab, India; 3 Department of Biochemistry & Molecular Biology, The University of Texas Medical Branch at Galveston, Galveston, TX, United States; 4 Centre for Research Impact & Outcome, Chitkara College of Pharmacy, Chitkara University, Rajpura, Punjab, India; 5 Center for Innovation and Inclusive Research, Sharda University, Greater Noida, Uttar Pradesh, India; 6 Centre for Experimental Pharmacology & Toxicology, Central Animal Facility, JSS Academy of Higher Education & Research, Mysuru, Karnataka, India; 7 Department of Pharmacology, JSS College of Pharmacy, JSS Academy of Higher Education & Research, Mysuru, Karnataka, India; 8 Institute for Integrated Cell-Material Sciences (WPI-iCeMS), A210, Kyoto University Institute for Advanced Study, Kyoto, Japan

**Keywords:** calcium homeostasis, gene expression dynamics, mitochondrial bioenergetics, reactive oxygen species, sleep regulation

## Abstract

Sleep is an essential biological function with unclear mechanisms. Recent research links the circadian clock, energy metabolism, calcium handling, reactive oxygen species (ROS) production, and synaptic plasticity to cognitive performance. Given that mitochondria regulate cellular energy production, calcium buffering, and ROS dynamics, it stands to reason that mitochondrial function plays a significant role in sleep regulation. Here, we propose an enhanced integrative framework “the mitochondrial theory of sleep” that synthesizes disease models, mechanistic details, and gene enrichment analysis based on bioinformatics. We identified nine crucial mitochondrial protein-coding genes that are highly enriched in pathways linked to oxidative phosphorylation, synaptic processes, and sleep characteristics by closely analyzing data sets using Gene Ontology, KEGG, and Reactome databases. Our analysis suggests that sleep deprivation is associated with calcium signaling, mitochondrial genes expression, reactive oxygen species (ROS) generation, and ATP production. Additionally, coherent mitochondrial genes cluster linked to neuroprotective and sleep-inducing effects are revealed by functional clustering and network topology analysis. This study offers a testable model for the control of wakefulness and sleep by the mitochondria. This mechanistic model incorporates findings from research on humans and animals, psychiatric conditions including bipolar disorder, and evolutionary theories. This framework identifies promising therapeutic targets for future investigation, including (MCU1, CPT1A, and antioxidant regulators) to treat sleep disorders in addition to bringing disparate findings together from a biological perspectives. While much of the current evidence remains correlative, this framework provides a roadmap for future studies. By highlighting the function of mitochondrial dynamics as an integrating point for metabolic and neurophysiological mechanisms of sleep, our findings advance our theoretical and practical understanding of sleep.

## 1 Introduction

“Any direct expression of a fact in science is valuable as temporary generalities and schemes, which are at times necessary for preliminary navigate theory, but it needs to be always checked or corrected when a rule which has become a scheme, proves inadequate.”-Julius Sachs, ‘Fabric and Shape of Plant Organs’, 1880.

Although sleep is a basic biological process found in almost all animal species, its fundamental function and underlying mechanisms remain poorly understood. Many theories that explain sleep from various functional perspectives have been put forth over the years. These include adaptive inactivity (1), energy conservation (2), restorative processes for brain and body (3–5), immune support (6), and significantly impaired thermoregulation (7, 8), particularly during rapid eye movement (REM) sleep (9). Sleep has also been linked to developmental and maturational needs (10–12), synaptic and neuronal plasticity (13), the restoration of brain energy metabolism (3), and the clearance of neurotoxins via the glymphatic system (14). For further information on the functions of sleep, readers are encouraged to consult several comprehensive reviews (15–19).

Additionally, sleep is essential for numerous cognitive and emotional processes, including mood regulation (20), learning (21), memory processing, reactivation and consolidation (22–28), executive function (29), emotional integration (28), and broader information processing and brain reconfiguration (30, 31). Collectively, these findings underscore the critical role of sleep in maintaining both physiological stability and cognitive function (21, 32, 33). Despite the diversity of existing sleep models, most focus on specific physiological outcomes and do not adequately explain sleep at the cellular level. Consequently, few attempts have integrated the molecular mechanisms that could unify these diverse functions, highlighting the need for a comprehensive molecular biology model of sleep. We propose that mitochondrial activity may represent a central component of such a framework, supported by the recent findings of Sarnataro et al., which provide the first direct evidence linking sleep regulation to mitochondrial function (34).

The phosphorylation hypothesis of sleep posits that wake-related synaptic firing results in the accumulation of phosphorylated synaptic proteins and suggests that sleep restores a balance (35). Similarly, slow-wave oscillations in NREM sleep are accounted for the calcium signaling hypothesis, which relies calcium entry results in cortical membrane hyperpolarization. In turn, to reinitialize cortical activity, calcium-dependent kinases modulate pump activity and ion channels (36–39). These mechanisms are intrinsically linked to mitochondrial function, as mitochondria are the primary regulators of cellular calcium buffering and ATP-dependent kinase activity.

Mitochondria may be important for both NREM and REM sleep. Cellular repair, synaptic downscaling, and energy storage (ATP; Adenosine Triphosphate) are associated with NREM sleep and require functional oxidative phosphorylation (OXPHOS). On the other hand, REM sleep is associated with increased brain metabolism, heat production, and neurochemical synthesis, all of which are dependent on mitochondrial function. The roles of the various stages of sleep are different but related. The slow-wave activity (SWA) of NREM sleep is linked to synaptic pruning and downscaling, which necessitate the replenishment of cerebral energy stores like ATP, according to the Synaptic Homeostasis Hypothesis (3, 13). Molecular evidence supports sleep’s significance in promoting energy-dependent mechanisms that support synapses (40). REM sleep, on the other hand, has a unique metabolic and neuromodulatory environment. It is linked to mitochondrial thermogenesis to sustain its distinct neural activity, which may be essential for cellular function and signalling (41), as well as increased production and turnover (42). These factors suggest that mitochondrial function may be a unifying metabolic substrate for explaining the physiological functions of both phases of sleep. Current hypotheses about many aspects of sleep, such as the glymphatic system activity and synaptic homeostasis, often overlook the contribution of mitochondria.

There is now growing evidence that mitochondrial dysfunction may be a key mechanistic link between impaired sleep and neuroinflammation/cognitive impairments and neurodegenerative pathology. Experimental and clinical studies of sleep disturbances over the last few years have shown that disturbed sleep can affect both central and peripheral mitochondrial bioenergetics, oxidative stress pathways, inflammatory signaling, and mitochondrial quality control pathways. In animal models, sleep fragmentation activates FKBP51-mediated NF-kappaB signaling, resulting in mitochondrial respiratory chain failure, accumulation of reactive oxygen species, and hippocampal neuroinflammation, which can be corrected by pharmacologically inhibiting the action of FKBP51, thereby restoring mitochondrial integrity (43). Further, insomnia models demonstrate that complementation of mitochondrial homeostasis (mitochondrial membrane potential stabilization, recovery of the oxidative phosphorylation, and improvement in ATP production) can markedly ameliorate sleep disturbances and anxiety-like behavior (44). Importantly, clinical evidence in chronic insomnia disorder patients have revealed that the levels of circulating regulatory proteins of mitochondria (SIRT1 and SIRT3) are altered, showing that a relationship exists between sleep quality, thus providing translational support for the involvement of mitochondria in human sleep disorders (45).

Apart from bioenergetic abnormalities, other potential processes that may contribute to sleep-related neurological disorders include altered mitochondrial dynamics, mitophagy, calcium homeostasis, and respiratory chain deficiencies (46), such as Miro1 signaling and Complex III activity defects (47). Collectively, these findings offer persuasive evidence that mitochondrial dysfunction is both a result of insufficient sleep and a substantial factor in sleep pathology and its neurological repercussions. These factors suggest that mitochondria-targeting strategies warrant further investigation as potential therapeutic approaches. In this review, we synthesize evidence from molecular biology, clinical studies, and bioinformatics to propose a unified framework. We do not claim that mitochondrial dysfunction is the sole cause of sleep disturbances; rather, we argue that mitochondrial function serves as an integrating hub as a common pathway through which diverse physiological and pathological processes influence sleep.

## 2 Mitochondrial functions relevant to sleep

Mitochondria are highly dynamic and energy-producing organelles, often referred to as the “cell’s powerhouse” because of their central role in ATP synthesis through oxidative phosphorylation (OXPHOS), the universal energy currency required for cellular activity (48, 49). Morelli et al. (2025) proposed that myelin functions as a proton capacitor, storing energy as protons during sleep and converting it into ATP via OXPHOS during wakefulness (50); however, this hypothesis should be interpreted cautiously in the context of more established mitochondrial and metabolic evidence. As the primary source of cellular energy, mitochondria are particularly critical in metabolically demanding tissues such as the brain, where efficient ATP production supports neuronal function and overall physiological homeostasis (48, 49).

Beyond energy production, mitochondria participate in numerous metabolic and signaling pathways that regulate behavior, whole-body physiology, immune signaling, steroidogenesis, thermogenesis, calcium homeostasis, redox regulation, apoptosis, fatty acid oxidation, the tricarboxylic acid (TCA) cycle, heme synthesis, and stem cell regulation (48, 51). The mitochondrial redox environment plays a pivotal role in oxidative signaling, modulating neuronal excitability and neurotransmitter synthesis, both of which are essential for sleep–wake regulation (52). Although Kempf et al. primarily focused on the redox regulation of voltage-gated potassium channels and the limited evidence linking mitochondria to sleep (52), subsequent studies have strengthened this association (34, 42, 53). In particular, Sarnataro et al. demonstrated that mitochondrial morphology and functional plasticity vary across animal species and physiological states, including sleep (34, 42). Consistent with these findings, mitochondrial dysfunction has been associated with sleep disorders, with reduced mitochondrial activity reported in insomnia (42, 54, 55) and impaired mitochondrial function implicated in the neurocognitive and cardiovascular consequences of obstructive sleep apnea (OSA) (56). **Table 1** summarizes the pleiotropic mitochondrial functions relevant to sleep health.

**Table 1:** Pleiotropic functions of mitochondria are central to optimal sleep health.

Mitochondrial-related functions	Evidence(s)	PubMed references
Energy metabolism	Prolonged wakefulness affects the brain’s energy and neurophysiology. Sleep refills the brain’s energy supplies, which have been drained during waking. Sleep deprivation causes oxidative stress and ATP depletion.	Benington and Heller, 1995 (3); Scharf et al., 2008 (57); Sarnataro et al., 2025 (34).
Redox balance	Sleep deprivation causes altered redox homeostasis.	Trivedi et al., 2017 (58); Richardson and Mailloux, 2023 (41).
Reactive oxygen species (ROS)	Chronic sleep loss or sleep deprivation results in oxidative stress. Sleep deprivation alters the activity of antioxidant enzymes.	Lima et al., 2014 (59); Lacedonia et al., 2015 (60); Reimund, 1994 (61); Hill et al., 2018 (62); Kempf et al., 2019 (52); Vaccaro et al., 2020 (63); Neculicioiu et al., 2023 (64).
Ca2+ regulation	Sleep deprivation impairs calcium signaling. Retaining mitochondrial calcium in the heart during sleep in mice shows a reduction in respiratory activity, dissipates membrane potential, and elevation of ROS levels. These changes may enhance vulnerability to cardiac stress as they go from wakefulness to sleep	de Souzaet al., 2012 (65); Bhosale et al., 2017 (66); Abdel- Ingiosi et al., 2020 (67); Rahman et al., 2021 (68).
Apoptosis	A 24-hour sleep deprivation causes an increase in BAX (BCL2 associated X), a pro-apoptotic protein, and decreased BCL2 (B-cell lymphoma-2), an anti-apoptotic protein. Selective REM sleep deprivation triggers neuronal apoptosis mediated by NE acting on α1 adrenoceptor and by triggering the mitochondrial intrinsic pathway.	Montes-Rodríguez et al., 2009 (69), Somarajan et al., 2016 (70).

## 3 Evidence linking mitochondria and sleep disorders

### 3.1 Mitochondrial dysfunction in bipolar disorder and sleep disturbances

Because they produce ATP, regulate redox balance, and buffer calcium, mitochondria are essential for maintaining brain health. Consequently, neuropsychiatric disorders are significantly impacted by their malfunction. Mitochondrial dysfunction has been identified as a significant etiological cause of bipolar disorder (BD), a disease characterized by mood instability, sleep issues, and cognitive deterioration. The onset and course of bipolar disorder are directly influenced by mitochondrial genetics, bioenergetics, and metabolic processes, according to the mitochondrial theory of the disorder (71, 72).

Patients with bipolar disorder often exhibit genetic and biochemical abnormalities in mitochondrial activity. Among these include decreased ATP production, increased oxidative stress, damaged mitochondrial DNA (mtDNA), and disrupted electron transport chain (ETC) activity (73). Furthermore, BD patients frequently experience sleep problems, which have been mechanistically linked to impaired mitochondrial signaling. These problems include irregular circadian cycles, insomnia, and hypersomnia.

There is a clear link between mitochondrial malfunction and sleep problems, as Han et al. showed that individuals with decreased mitochondrial DNA copy number, a measure of mitochondrial dysfunction, had significantly lower sleep quality (74). Circadian rhythm abnormalities in BD may be caused by mitochondrial signaling issues, which interfere with the synchronization of molecular clocks that regulate the sleep/wake cycle, according to another study (75). This is supported by the fact that insomnia and other sleep disorders disproportionately impact those with concurrent BD and mitochondrial dysfunction (76). Emerging evidence identifies MICU1 (mitochondrial calcium uniporter regulator 1), a key regulator of mitochondrial calcium homeostasis, as a candidate gene linking bipolar disorder (BD) and sleep through its effects on ATP production, oxidative stress, and neuronal excitability (66, 77).

The role of mitochondria in health, disease, and aging is crucial. Mitochondrial dysfunction is thought to be a key cause of many health-related diseases (78). Conditions such as fibromyalgia, neuropathic pain, migraine headaches, ataxia, transient ischemic attack, ischemia/reperfusion (I/R), septic shock, cardiomyopathy, coronary artery disease (CAD), chronic fatigue syndrome (CFS), epilepsy, stroke, ataxia, retinitis pigmentosa (RP), diabetes mellitus (T2DM), hepatitis C (Hep C), primary biliary cirrhosis (PBC)), mental disorders (e.g., depression, bipolar disorder, schizophrenia), and neurodegenerative (e.g., Parkinson’s disease (PD), Huntington’s disease (HD), dementia, and Alzheimer’s disease (AD) are associated with mitochondrial dysfunction (79–81). This dysfunctionality is known to be induced by various precursor conditions. These pathophysiological processes include, but are not limited to, elevated production of ROS, enhanced mitochondrial inducible nitric oxide synthase (NOS) activity, enhanced NO production, decreased respiratory complex activity, impaired ETC system, and opening of mitochondrial permeability transition pore (MPTP), and accumulation of damage to mtDNA, all of which ultimately result in mitochondrial dysfunction (82). Many of these conditions are comorbid with sleep disturbances, underscoring the pervasive influence of mitochondrial health on neurophysiological stability and reinforcing the plausibility of a mitochondria-centered theory of sleep regulation.

In summary, the converging evidence of mitochondrial dysfunction, circadian disruption, and sleep disturbances in BD strongly supports the mitochondrial framework. While the directionality of these relationships remains to be fully elucidated, these findings suggest that mitochondria may represent a common pathway through which mood and sleep are linked, offering potential avenues for novel therapeutic strategies that target bioenergetic pathways.

### 3.2 Sleep deprivation and mitochondrial alterations

Sleep deprivation significantly affects mitochondrial function, leading to a number of structural and metabolic abnormalities. For example, sleep deprivation changes the expression of mitochondrial genes, raises oxidative stress, and modifies the architecture of organelles in all species, particularly in the energy-intensive areas of the brain (34, 42). According to Vaccaro et al., sleep deprivation is linked to mitochondrial dysfunction, which is marked by enhanced oxidative stress indicators and a higher capacity for the formation of reactive oxygen species (ROS) in a variety of tissues (63). Reduced ATP synthase activity, reduced amounts of mitochondrial proteins, and reduced mitochondrial density in neurons are the causes of these dysfunctions. The ensuing bioenergetic failure raises oxidative stress, which impairs cognitive function and cellular resilience. The ATP-producing enzyme mitochondrial ATP synthase may also become less active as a result of sleep deprivation (34, 83).

The reduced ATP generation may impair cellular activity. For example, Drosophila dorsal fan-shaped body (dFB) neurons switch from the hyperkinetic to the NADP+-bound form in response to an increase in mitochondrial ROS brought on by sleep deprivation. By increasing the frequency of action potentials and reducing the inactivation of the A-type current, the oxidation of the cofactor facilitates sleep (52).

Sleep deprivation (SD) causes endoplasmic reticulum (ER) stress, which is characterized by increased interaction between the ER and mitochondria, according to research by El Alaoui and colleagues (84). According to their research, SD was associated with notable transcriptional changes in the forebrains of mice related to ER stress-related genes, ER-mitochondria interaction, calcium homeostasis, and mitochondrial respiratory activity. For example, an electron microscopy (EM) study revealed that ER cisternae formed new contact sites with mitochondria called mitochondria-associated membranes (MAMs), which are crucial hubs for the transport of chemicals like calcium and lipids as well as for regulating redox status and ATP synthesis. When combined, these findings demonstrate that sleep deprivation interferes with signaling pathways, organelle crosstalk, and mitochondrial energetics, all brain functions that control sleep-wake cycles.

Lack of sleep can lead to significant changes in mitochondrial activity, which can then negatively impact brain health and eventually sleep regulation. According to recent studies, paradoxical sleep deprivation, i.e., not getting enough REM sleep, increases brain ammonia, which boosts mitochondrial glutamate synthesis through the activity of α-ketoglutarate dehydrogenase (α-KGDH), connecting nitrogen metabolism to mitochondrial adaptations during sleep loss (85). This study demonstrates how sleep deprivation affects signaling pathways, organelle communication, and mitochondrial energetics,all of which are critical brain functions for sleep-wake regulation.

### 3.3 Mitochondria-associated membranes: a critical interface

Mitochondria-associated membranes (MAMs) are specialized contact sites between the endoplasmic reticulum (ER) and mitochondria that serve as dynamic signaling platforms integrating calcium homeostasis, lipid metabolism, and redox signaling (86–88). Rather than functioning as isolated organelles, the ER and mitochondria communicate through MAMs to coordinate cellular bioenergetics, metabolic adaptation, and stress responses, processes increasingly recognized as fundamental to sleep–wake regulation (42). Recent evidence demonstrates that sleep deprivation remodels these ER–mitochondria contact sites, suggesting that MAMs are key mediators of the cellular response to prolonged wakefulness (84).

A principal function of MAMs is the regulation of calcium transfer from the ER to mitochondria through the coordinated action of inositol 1,4,5-trisphosphate receptors (IP3Rs), voltage-dependent anion channels (VDACs), and the mitochondrial calcium uptake machinery (89–91). Mitochondrial calcium uptake stimulates tricarboxylic acid cycle dehydrogenases and oxidative phosphorylation, thereby matching ATP production with neuronal energy demands (49, 92). This mechanism closely aligns with the Ca²^+^-dependent hyperpolarization hypothesis of sleep, in which calcium dynamics regulate neuronal excitability and sleep homeostasis (36–38, 93). Supporting this concept, Aboufares El Alaoui et al. (2023) demonstrated that sleep deprivation increases both the number and size of MAM contacts, enhances ER-to-mitochondria calcium transfer, and alters the expression of MAM-associated proteins involved in mitochondrial respiration and calcium homeostasis (84).

Beyond calcium signaling, MAMs are major hubs for phospholipid synthesis and trafficking required for mitochondrial membrane integrity and respiratory chain assembly (94–96). They also facilitate bidirectional reactive oxygen species (ROS) signaling between the ER and mitochondria, integrating oxidative stress with cellular metabolism (97, 98). Consistent with these functions, sleep deprivation has been associated with enhanced ER stress, increased oxidative damage, and mitochondrial dysfunction (63, 64, 99). Furthermore, mitochondrial metabolic adaptations, including alterations in the α-ketoglutarate dehydrogenase (α-KGDH)/ammonia pathway, have been reported during prolonged wakefulness (85), reinforcing the concept that mitochondrial metabolism dynamically responds to sleep pressure.

Collectively, MAMs represent a critical but understudied nexus in the mitochondrial theory of sleep. Their regulation of calcium signaling directly links to the Ca²^+^-dependent hyperpolarization hypothesis, while their role in lipid metabolism connects to membrane remodeling during synaptic homeostasis (100). Future studies investigating MAM dynamics across the sleep–wake cycle could provide crucial mechanistic insights into how mitochondrial signaling coordinates sleep regulation. These findings collectively illustrate a vicious cycle: sleep deprivation impairs mitochondrial function, which in turn may exacerbate sleep-wake dysregulation. This bidirectional relationship is a core prediction of the mitochondrial framework.

## 4 Mitochondrial genomic factors in sleep regulation

### 4.1 Mitochondrial DNA as a regulator of sleep/wake function

Thirteen essential proteins found in mitochondrial DNA (mtDNA) are involved in ATP generation via OXPHOS (101). A living indicator of mitochondrial health, mitochondrial DNA (mtDNA) is inherited from the mother, exists in numerous copies per cell, and is especially susceptible to oxidative stress (102). Emerging evidence suggests that mitochondrial DNA (mtDNA) integrity and variation may influence sleep-wake regulation. However, it is important to note that most of these findings are correlative, and the relationship between mtDNA mutations and sleep phenotypes requires further experimental validation.

Mice with a mutation in their mtDNA that caused ATP generation malfunction in a seminal experiment displayed abnormal circadian patterns, including increased waking during the day, decreased sleep onset latency (SOL), and increased vigilance throughout the night (103). This finding implies that circadian entrainment and sleep-wake regulation may be affected by mtDNA failure, which leads to a deficit in ATP, either through changes in neuronal excitability or energy-dependent communication in the brain.

This is corroborated by research on humans, which shows that specific mtDNA haplotypes are associated with an increased risk of insomnia and poor sleep quality. Affected people reported having trouble falling and maintaining asleep, as well as waking up in the evening (104). These correlations imply that individual vulnerability to sleep disorders may be influenced by genetic variations in mtDNA.

Although the underlying mechanisms of these effects are unknown, these findings suggest that mtDNA is a metabolic modulator as well as a possible modulator of circadian and sleep-wake rhythms. More research is needed to understand the effects of mtDNA integrity, copy number, and mutation burden on sleep physiology as well as the possible advantages of treating sleep disorders by focusing on mtDNA stability.

### 4.2 Mitochondrial genes associated with sleep and their functional characterization

Among the 13 protein-coding genes encoded by the human mitochondrial genome, nine genes were identified as being associated with sleep: ND1, ND2, ND4, ND5, COX1, COX2, COX3, ATP6, and MT-CYB. These genes encode essential subunits of the mitochondrial electron transport chain (ETC), including Complex I (ND1, ND2, ND4, and ND5), Complex III (MT-CYB), Complex IV (COX1, COX2, and COX3), and Complex V (ATP6). Their distribution across four respiratory chain complexes underscores the importance of mitochondrial bioenergetics in sleep-related physiological processes and suggests that alterations in ATP production and oxidative metabolism may represent common molecular mechanisms underlying sleep regulation. **Table 2** summarizes the associations between the nine mitochondrial protein-coding genes and a range of sleep-related as well as non-sleep-related disorders, highlighting their potential roles in both physiological and pathological processes.

**Table 2:** Association of 9 mitochondrial protein-coding genes with sleep-related and non-sleep related disorders.

Gene	Full name	Description of the gene associated with sleep	Associated disorder	References
ND1	NADH dehydrogenase, subunit 1 (complex I)	In monozygotic twin pairs, the amount of mtDNA copy number fluctuates based on habitual variances in sleep duration	Bipolar disorder	Wrede et al., 2015 (105)
ND2	MTND2	Studies have shown that during spontaneous wakefulness in the brain of mice, there is a surge in the mRNA and protein levels of the mitochondrial ETC. This includes complex IV subunits, which encode cytochrome c oxidase I (COX1) and cytochrome c oxidase subunit II (COX2), and complex I subunit that encodes NADH dehydrogenase 2 (ND2) subunits.	Reduced eye contact	Nikonova et al., 2010 (106)
COX1	Cytochrome c oxidase subunit I	Studies have shown that during spontaneous wakefulness in the brain of mice, there is a surge in the mRNA and protein levels of the mitochondrial ETC. This includes complex IV subunits, which encode cytochrome c oxidase I (COX1) and cytochrome c oxidase subunit II (COX2), and complex I subunit that encodes NADH dehydrogenase 2 (ND2) subunits.	Primary Myocardial Diseases	Nikonova et al., 2010 (106)
COX2	cytochrome c oxidase subunit II	The comparison between obese children with Obstructive Sleep Apnea-Hypopnea Syndrome (OSAS) and non-obese children reveals the expression of LXR, cholesterol ester transfer protein (CETP), and cyclooxygenase-2 (COX-2) genes	Depressive disorder	Ye et al., 2017 (107)
ATP8	ATP synthase F0 subunit 8	An 8-year-old Caucasian boy was treated with valproate medication for two years. The EEG abnormalities, neuropsychological deficits, along with well-pronounced irritability and learning deficits were reported.	Cardiomyopathy	Ye et al., 2017 (107)
ATP6	ATP synthase F0 subunit 6	A well-established and characterized Drosophila model of mitochondrial encephalomyopathy (ATP6 genetic line) shows sleep disruption	Schizophrenic disorders	Fogle et al., 2019 (108)
ND5	NADH dehydrogenase, subunit 5 (complex I)	Among UK Biobank subjects with two severity categories of depression, genome-wide association studies (GWAS) including eight accelerometry-derived sleep metrics were conducted across both the autosomal and mitochondrial DNA (mtDNA). Concerning sleep, MT-ND2 and MT-ND5 are potential candidate genes.	MELAS syndrome	Beaupre et al., 2023 (104)
ND6	NADH dehydrogenase, subunit 6 (complex I)	Through glucocorticoid receptors, corticosteroids and stress control the expression of the mtDNA gene in rat hippocampus	MELAS syndrome	Kokkinopoulou and Moutsatsou, 2021 (109)
CYTB	Cytochrome b	Differences in mtDNA influences Drosophila melanogaster locomotor activity and sleep/wake cycle	Colorectal Cancer	Anderson et al., 2022 (110)

To investigate whether these genes constitute a functionally coherent network rather than a random subset of mitochondrial genes, we performed functional enrichment analysis using PESCADOR. This analysis integrates Gene Ontology (GO), Kyoto Encyclopedia of Genes and Genomes (KEGG), and Reactome pathway annotations to identify biological processes and molecular pathways that are significantly overrepresented among the selected genes relative to the mitochondrial genome background. The enrichment results demonstrated a remarkable convergence on pathways involved in mitochondrial energy metabolism, indicating that the identified sleep-associated genes participate in highly interconnected bioenergetic functions rather than isolated cellular processes.

**Figure 1:**
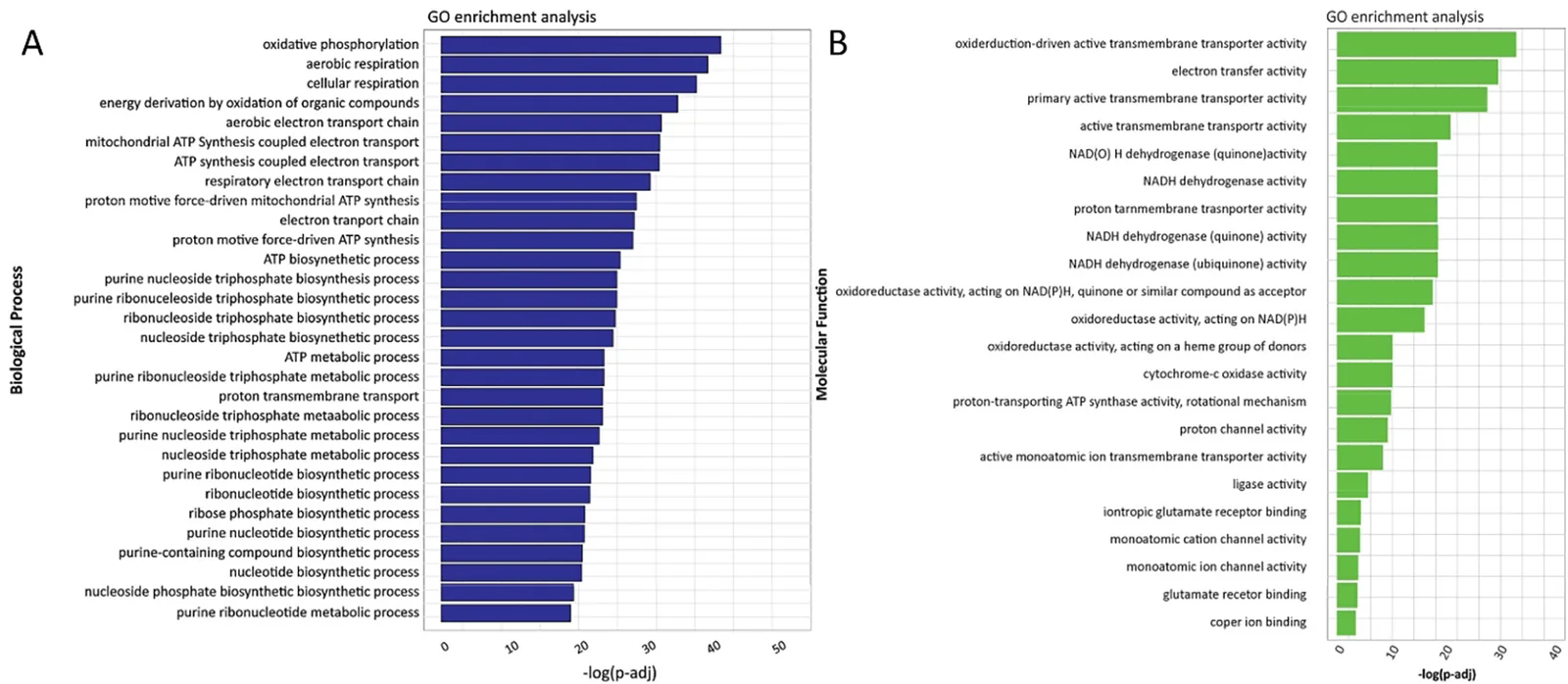
GO enrichment analysis of mitochondrial genes. **(A)** Top enriched biological processes related to oxidative phosphorylation, ATP synthesis, and metabolism. **(B)** Top enriched molecular functions involving transmembrane transporter, electron transfer, and oxidoreductase activities. Bars represent –log10(adjusted p-values); GO terms were identified using ClusterProfiler with Benjamini–Hochberg correction (adjusted p < 0.05).

**Figure 2:**
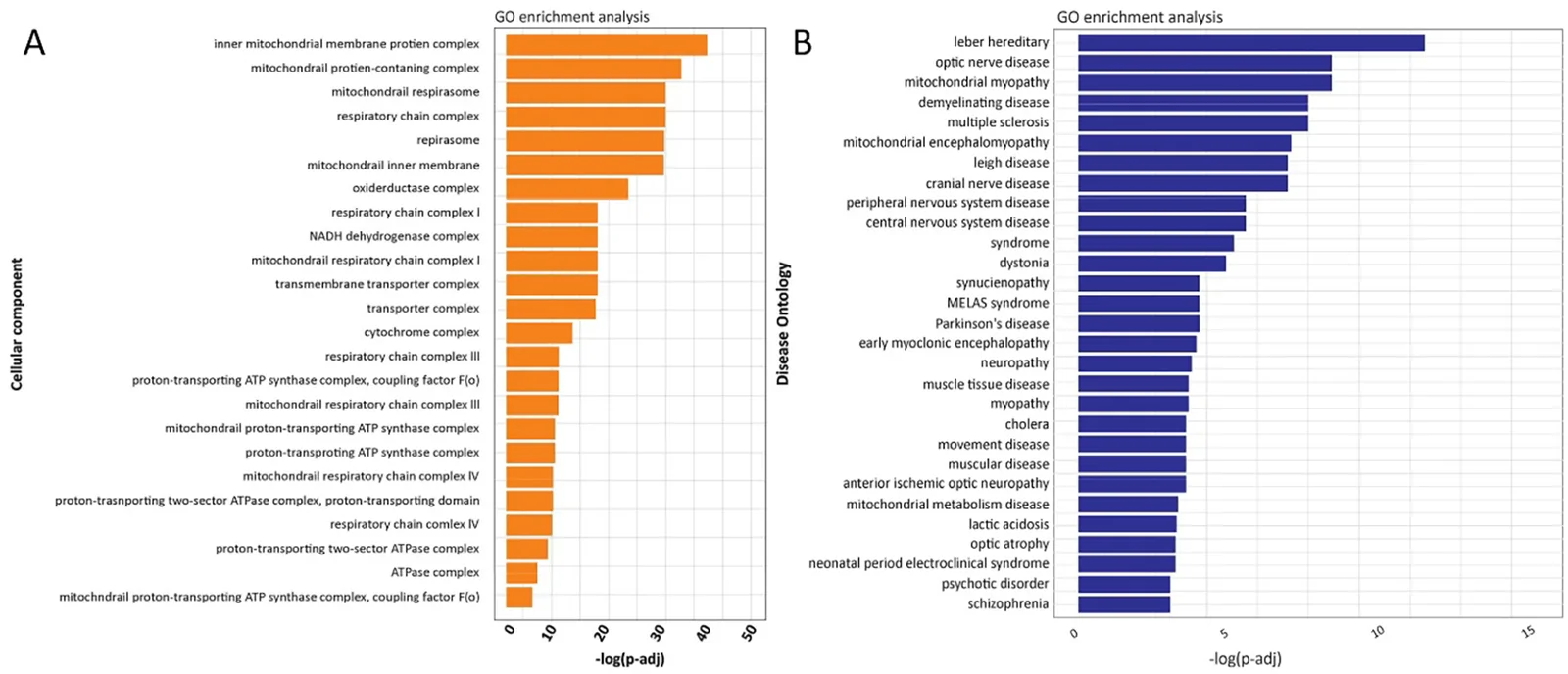
GO and disease ontology enrichment analysis of mitochondrial genes. **(A)** Top enriched cellular components, including mitochondrial protein and respiratory chain complexes. **(B)** Disease ontology terms associated with mitochondrial and neuro-metabolic disorders. Bars represent –log10(adjusted p-values); enrichment reflects disease annotations of the input gene set.

**Figure 3:**
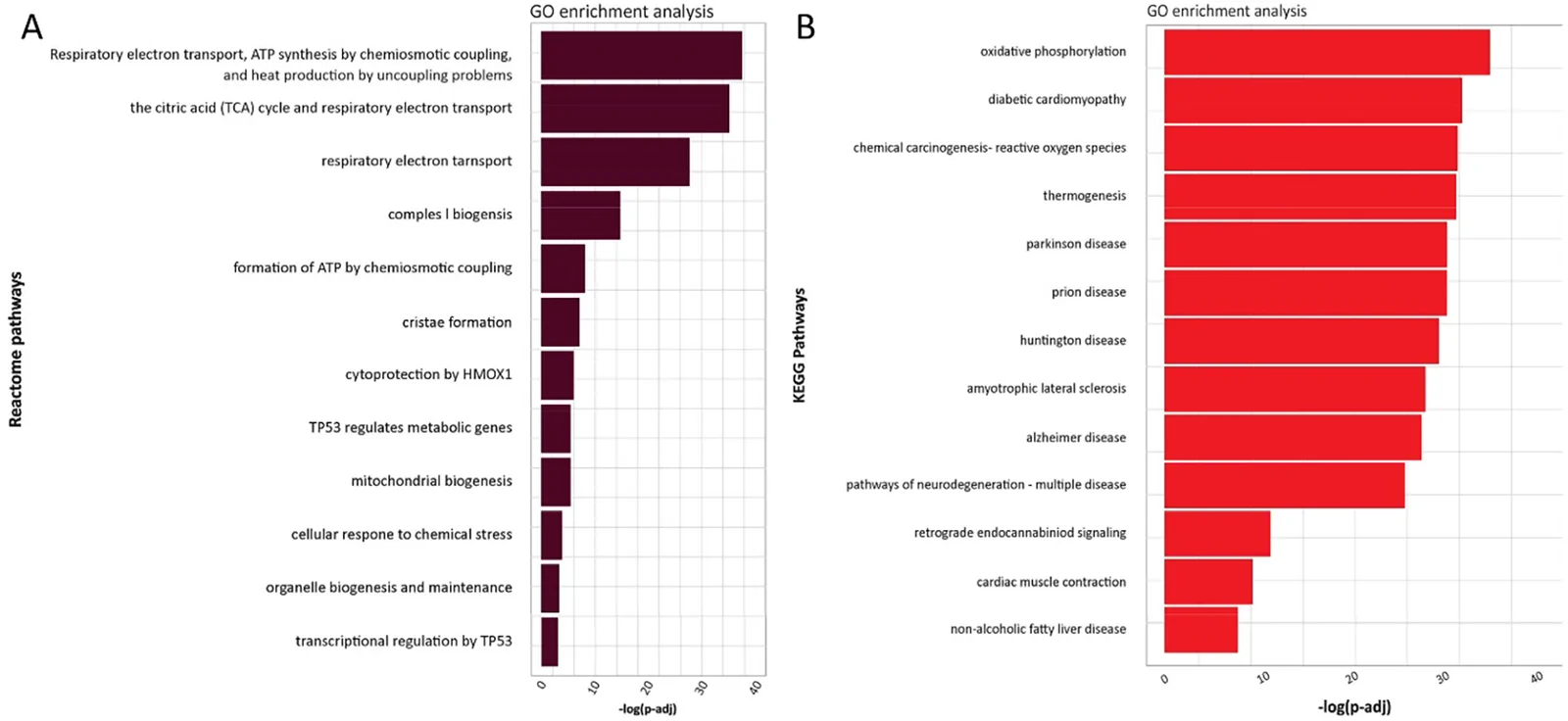
Reactome and KEGG pathway enrichment of mitochondrial genes. **(A)** Reactome pathways related to respiratory electron transport, ATP synthesis, and mitochondrial biogenesis. **(B)** KEGG pathways associated with oxidative phosphorylation, neurodegenerative diseases, and metabolic disorders.

KEGG pathway analysis revealed oxidative phosphorylation as the most significantly enriched pathway, together with neurodegenerative disease pathways such as Parkinson’s disease, which share common defects in respiratory chain Complex I and oxidative phosphorylation components (**Figure 1**). Similarly, Reactome pathway enrichment highlighted respiratory electron transport, ATP synthesis by chemiosmotic coupling, and mitochondrial translation, emphasizing the coordinated role of these genes in maintaining mitochondrial respiratory efficiency and ATP production (**Figure 2**). Gene Ontology enrichment further demonstrated significant overrepresentation of biological processes related to cellular respiration, electron transport chain activity, proton motive force generation, oxidative phosphorylation, and ATP metabolic processes, confirming that these genes collectively function within the core mitochondrial energy-producing machinery (**Figure 3**).

**Figure 4:**
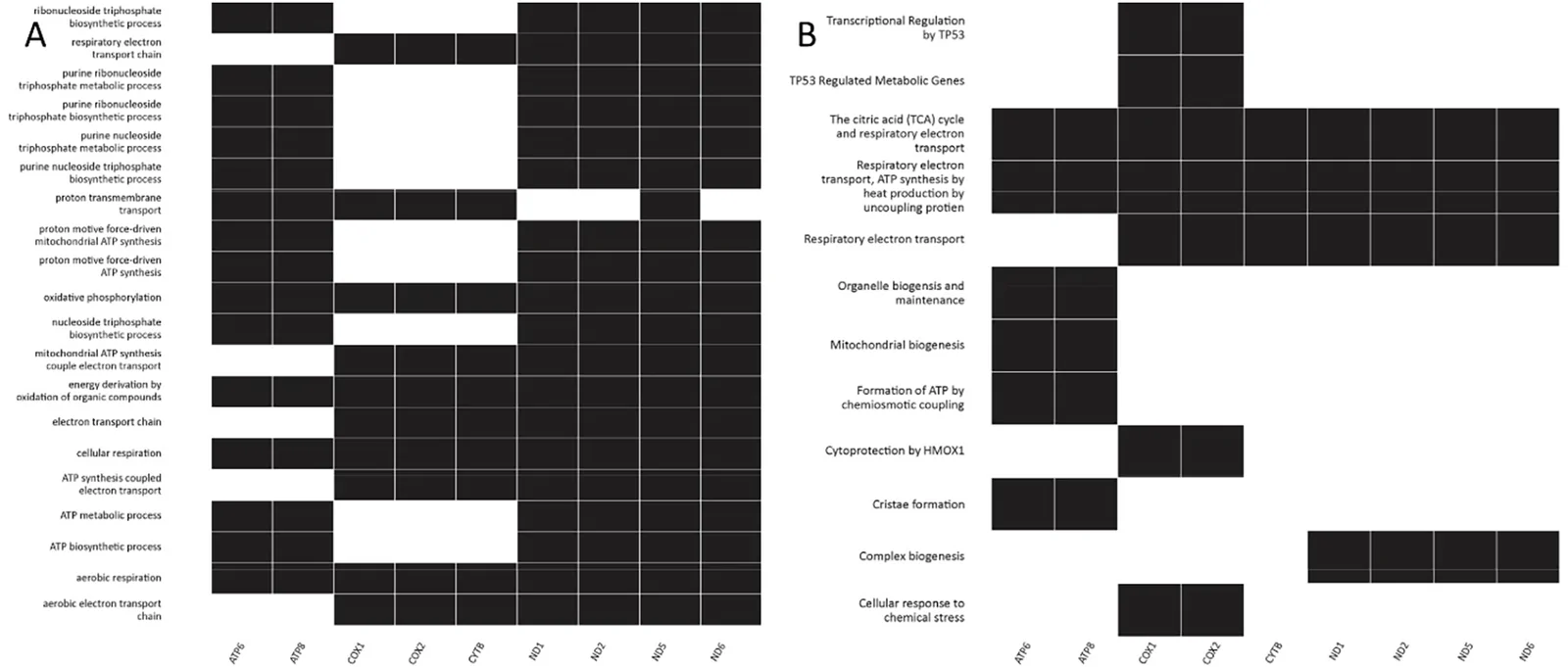
Functional enrichment of TP53-regulated mitochondrial genes. **(A)** Metabolic, ATP biosynthetic, and mitochondrial processes. **(B)** Transcriptional regulation, TCA cycle, electron transport, and mitochondrial biogenesis.

**Figure 5:**
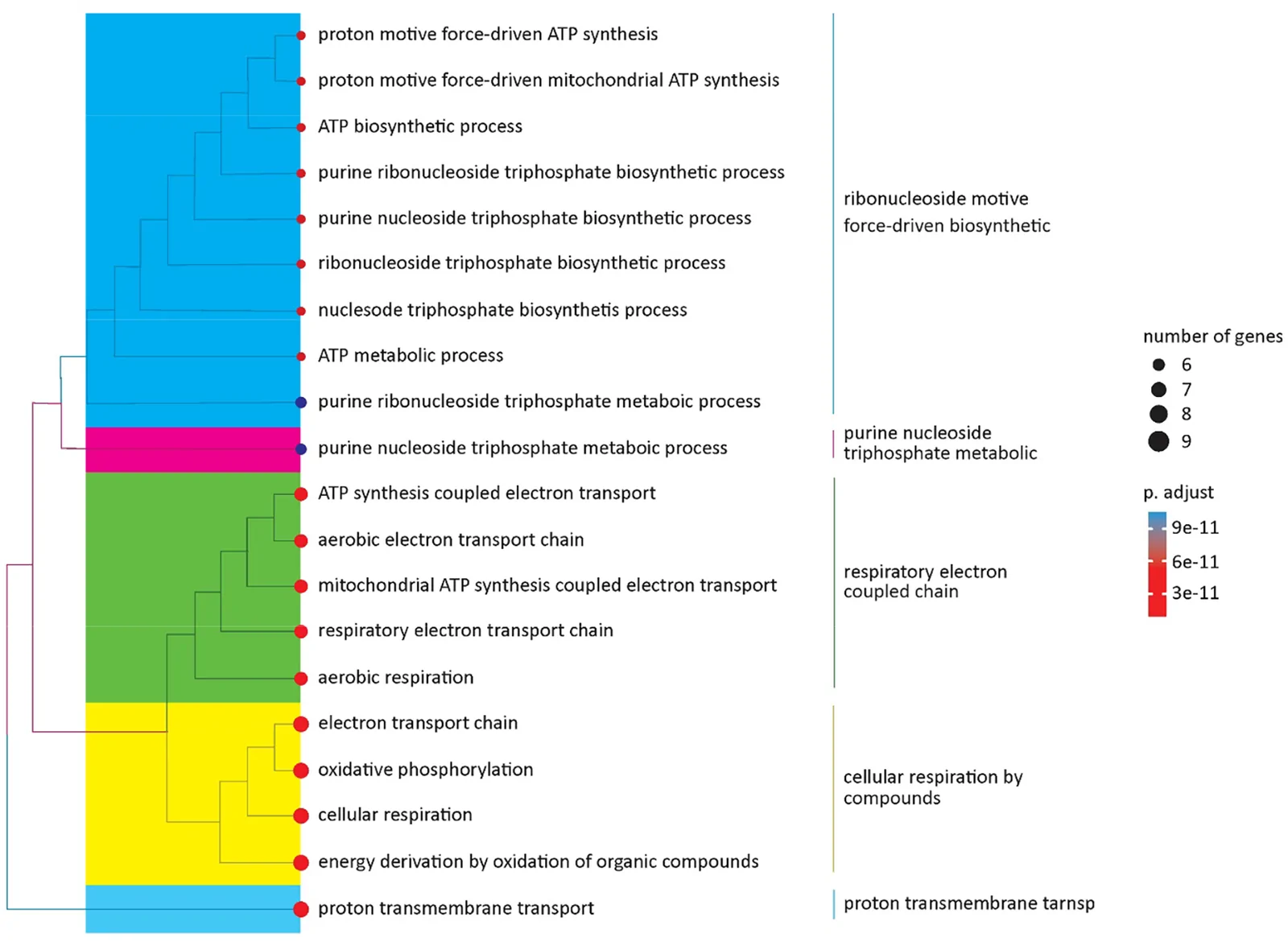
Hierarchical clustering of GO terms related to mitochondrial and metabolic processes. Dot size indicates gene counts, while color represents adjusted p-values.

**Figure 6:**
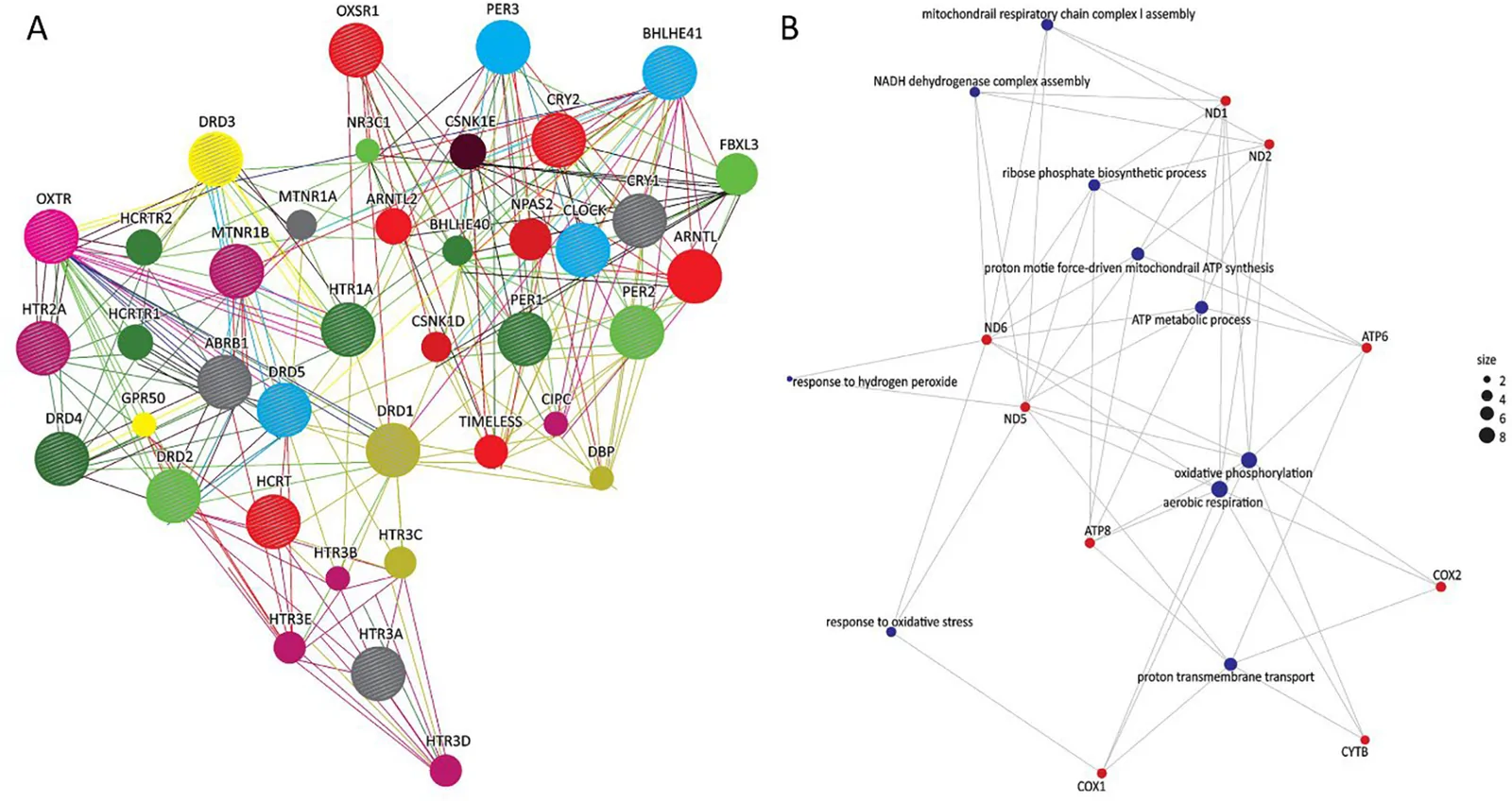
Gene interaction and functional enrichment networks. **(A)** Circadian rhythm-related gene interaction network. **(B)** GO network of mitochondrial processes, highlighting enriched functional connections.

To further examine functional relationships among the identified genes, network analysis was performed, revealing that ND1, COX1, and ATP6 occupy central positions within the interaction network and connect multiple enriched pathways, suggesting that they act as major functional hubs coordinating mitochondrial bioenergetic activity (**Figure 4**). Hierarchical clustering of enriched functional categories further demonstrated coordinated organization of oxidative phosphorylation, respiratory electron transport, and ATP synthesis modules, highlighting the close functional relationship among these biological processes (**Figure 5**). Disease enrichment analysis additionally linked the identified genes to mitochondrial disorders, including Leber hereditary optic neuropathy and mitochondrial myopathy, both of which are characterized by impaired oxidative phosphorylation and have been associated with sleep disturbances (**Figure 6**). The detailed methodological information of bioinformatics analysis and detailed figure legends is presented in **Supplementary Methods**.

Collectively, these findings demonstrate that the nine sleep-associated mitochondrial genes are not only structurally distributed across the respiratory chain but are also functionally integrated within conserved pathways governing mitochondrial energy metabolism. The consistent enrichment of oxidative phosphorylation, electron transport, and ATP synthesis across GO, KEGG, and Reactome analyses provides a robust functional framework supporting the hypothesis that mitochondrial bioenergetic dysfunction represents a key molecular mechanism linking mitochondrial genetics to sleep regulation.

## 5 Mitochondria and sleep physiology

### 5.1 Mitochondria, reactive oxygen species, and sleep

Chemically reactive molecules known as reactive oxygen species (ROS) are mostly produced as byproducts of mitochondrial respiration. ROS are crucial signaling molecules, even though they are typically linked to oxidative stress and cellular damage. According to recent studies, ROS may influence memory development, sleep regulation, and neural plasticity (64, 99, 111).

The restorative function of sleep in preserving redox equilibrium and mitochondrial integrity is supported by animal studies showing that mitochondrial ROS typically build up during waking and are subsequently reduced during sleep (112). Moderate ROS levels may be required to promote cellular processes during sleep, such as synapse remodeling and metabolic clearance, rather than being solely harmful. While these findings are intriguing, it remains to be determined whether ROS are a cause, consequence, or both of sleep-wake regulation. The distinction between physiological and pathological ROS levels is critical for interpreting these results.

Clinical and experimental evidence further supports this role. Elevated brain ROS levels have been observed in individuals with mitochondrial dysfunction or metabolic disorders, correlating with impaired sleep quality and cognitive deficits (56, 113–115). A regulated redox environment is essential for restorative sleep, which, in turn, aids in the elimination of reactive oxygen species that accumulate during waking (112).

In addition to sleep regulation, ROS appears to influence memory consolidation and learning. For instance, studies in mice have shown that elevated ROS levels during sleep enhance performance on memory tasks, whereas reduced ROS levels impair learning (116). These results point to ROS’s dual function as metabolic byproducts and active modulators of sleep-dependent cognitive processes. Our gene enrichment analysis supports this link, as sleep-associated mitochondrial genes were significantly enriched in pathways related to oxidoreductase activity and electron transfer (**Figure 1B**), the processes that generate ROS. Future studies are required to distinguish between physiological and pathological ROS levels to identify potential antioxidant-based treatments for sleep disorders.

### 5.2 Mitochondrial diseases and sleep

Mitochondrial diseases (MDs) are a broad family of genetically inherited disorders that arise because of mutations in nuclear genes encoding proteins required for mitochondrial function or mitochondrial DNA (mtDNA). These diseases can affect multiple organ systems, especially the nervous system. This is because many neural and other tissues are highly dependent on aerobic metabolism inasmuch as mitochondria are present in every nucleated cell and are involved in the synthesis of ATP.

Sleep dysfunction is a common, yet underrecognized, symptom of mitochondrial diseases. Hypersomnia, interrupted sleep, dysfunctional rapid eye movement (REM) sleep, and altered circadian patterns are frequently reported in MDs (117). Changes in the central nervous system’s energy production, especially in brain regions such as the brainstem and hypothalamus that control arousal and circadian rhythm, may be the source of these symptoms.

Because of their wide range of symptoms and overlap with other neurological or metabolic disorders, MDs can be difficult to diagnose. In combination with symptoms including weakness, fatigue, and intolerance of exercise, disturbances of sleep in these patients may be an early indicator or additional diagnostic marker.

Understanding the role of sleep deprivation in mitochondrial disease may help target treatments. The effects of these disorders on sleep and quality of life may be lessened by enhancing mitochondrial activity with medications, genetic therapies, better sleep hygiene, and exercise. The frequent occurrence of sleep disturbances in MDs provides compelling clinical evidence for the mitochondrial theory. These observations suggest that sleep dysfunction in MDs is not a secondary symptom but a direct consequence of compromised energy metabolism in sleep-regulating brain regions. This supports the view of sleep as an energy-conserving and restorative state and highlights the potential of mitochondrial biomarkers for early detection of sleep problems in MD patients.

### 5.3 Mitochondria, energy metabolism, and sleep

Mitochondria are essential for cellular energy metabolism during sleep, when the brain’s energy needs and metabolic processes are dynamically controlled. Mitochondria affect circadian rhythms because they are both molecular clock effectors and regulators (118). The daily fluctuations of mitochondrial respiration are a reflection of an organism’s sleep-wake cycle. To assist in synchronizing physiological processes with ambient light/dark (LD) cues, for example, mitochondrial activity increases during active phases and decreases during sleep (118). Due to these consequences, a feedback loop is created whereby poor mitochondrial function deteriorates sleep quality, which further impairs mitochondrial function. Treatments aimed at improving mitochondrial health have demonstrated potential for improving the quality of sleep. Exercise has been demonstrated to improve respiratory efficiency and mitochondrial biogenesis, which improves the quality of sleep (119). Recently, Jiang et al. (2025) identified mitochondrial energy metabolism genes associated with obstructive sleep apnea syndrome through bioinformatics analysis, further supporting the link between mitochondrial function and sleep-disordered breathing (120–122). In addition to maintaining systemic metabolic balance, maintaining mitochondrial health is essential for controlling sleep architecture and circadian alignment (123). Collectively, these findings demonstrate that mitochondrial energy metabolism is not merely a passive consequence of sleep but an active participant in sleep-wake regulation and circadian alignment.

## 6 Genetic-level changes in mitochondria induced by sleep deprivation

Sleep loss modifies the profile of mitochondrial gene expression, which affects apoptosis, ROS levels, and energy balance (116, 124). Microarray study has shown that sleep-deprived mice had lower ATP production due to down-regulated components of the mitochondrial respiratory chain. It has been demonstrated that the overexpression of genes associated with oxidative stress, such as NADPH oxidase, increases ROS levels and mtDNA damage simultaneously (125). In addition to making breathing difficult, these changes increase the risk of neurological diseases and cognitive loss.

Genes that regulate fatty acid metabolism and mitochondrial biogenesis are similarly affected by sleep deprivation (126). For example, sleep deprivation causes a significant reduction in two important molecules that control mitochondrial energy production: carnitine palmitoyltransferase 1A (CPT1A) and peroxisome proliferator-activated receptor alpha (PPARα) (127, 128). Reduced mitochondrial activity, poor lipolysis, and enhanced glycolysis are the results of this disease.

The mitochondrial calcium uniporter (MCU) is of particular interest. MCU1 regulates mitochondrial calcium uptake, which is critical for ATP production and neuronal oscillatory activity. MCU1 is enriched in sleep-wake regions including the hypothalamus, thalamus, brainstem, and cortex (129). Mutations in MICU1, a regulator of MCU, have been linked to myopathy and extrapyramidal signs (77), and altered MCU function is associated with delayed sleep onset through reduced ATP availability (68, 77). These findings suggest MCU1 may be a molecular link between mitochondrial calcium handling, energy metabolism, and sleep-wake regulation.

Thus, sleep deprivation leads to dramatic changes in mitochondrial gene expression and function. This includes calcium dyshomeostasis, activation of oxidative stress response genes, and repression of energy-producing genes, leading to poor-quality sleep and chronic disease. Future research needs to identify genetic and epigenetic markers in sleep disorders of mitochondrial dysfunction to guide the development of personalized treatment. These genetic and molecular changes following sleep deprivation reveal multiple points of vulnerability in mitochondrial function that could be targeted for therapeutic intervention.

## 7 The rationale for the mitochondrial theory of sleep

The mitochondrial theory of sleep proposes that a primary function of sleep is to maintain mitochondrial integrity and function, and that mitochondrial activity regulates sleep demands, structure, and recovery. In contrast to other ideas that have limited their focus to cerebral, synaptic, or lymphatic mechanisms, This framework identifies mitochondria as an intersecting point intersecting point between metabolic homeostasis, neuronal excitability, and circadian regulation. This theory is supported by growing evidence that mitochondrial dysfunction affects several elements of sleep. People with sleep disorders have altered mitochondrial genes related to oxidative phosphorylation, calcium control, and ROS levels. For example, genetic differences in mitochondrial protein-coding and non-coding genes are linked to abnormal sleep-wake rhythms, insomnia, and hypersomnia. Transcriptome and functional imaging studies demonstrating alterations in mitochondrial bioenergetics in the brains of sleep-deprived individuals corroborate these conclusions.

Lastly, the main neurochemicals involved in sleep are regulated by mitochondria. They are involved in the production of neurotransmitters including GABA, dopamine, and melatonin that control circadian rhythm, arousal, and REM/NREM sleep (130, 131). One explanation for the fragmented sleep and irregular timing seen in mood disorders and neurodegenerative illnesses may be changes in the production of these molecules brought on by mitochondrial failure.

This framework offers several advantages. It offers a method for identifying biomarkers (such mitochondrial DNA haplogroups and ATP generation), creating treatments (like CoQ10 and MCU modulators), and comprehending individual variations in sleep susceptibility. Importantly, the hypothesis is in line with the systems biology approach, where there is no single mechanism regulating sleep, it is seen as the consequence of interactions between molecules, cells, and systems. The addition of metabolic and genetic information to behavioral data enriches our understanding of sleep and strengthens both the descriptive and predictive power of our sleep regulation models. Importantly, the mitochondrial framework does not replace existing theories but rather provides a metabolic substrate that unifies them. For instance, synaptic homeostasis requires ATP for protein synthesis and degradation; glymphatic clearance is energy-dependent; and immune function relies on mitochondrial signaling. Thus, the mitochondrial theory complements and deepens our understanding of these established models.

## 8 Discussion: toward a mitochondria-centric framework of sleep

“Nothing is more practical than a good theory.”— Ludwig Boltzmann

Numerous theories have been proposed to explain the adaptive functions of sleep, including its roles in network reorganization, memory consolidation, immune enhancement, detoxification, and energy homeostasis. Several of these theories emphasize mitochondrial energy metabolism. The free radical flux hypothesis proposes that sleep facilitates the removal of reactive oxygen species (ROS) generated during wakefulness, with studies demonstrating enhanced mitochondrial ROS clearance and repair during sleep (61). Similarly, the restorative theory suggests that sleep, particularly rapid-eye movement (REM) sleep, supports anabolic processes such as neuronal repair, protein synthesis, neurotrophic factor activation, and growth hormone release (4, 5, 132, 133). Complementing these concepts, the energy allocation theory posits that sleep redistributes energy from wakefulness toward essential cellular housekeeping functions, including tissue repair, immune regulation, and synaptic plasticity, while key metabolic intermediates such as ATP, glutamate, and lactate act not only as energy substrates but also as regulators of sleep architecture according to cellular energy status (128, 134, 135).

New evidence indicates that mitochondria are at the junction of these concepts. Mitochondria are not only the powerhouses of the cell (ATP), but also involved in calcium signaling, synthesis of neurotransmitters, redox balance, and circadian feedback. They play central roles in sleep physiology, as they are essential for synapse physiology, energy metabolism, and survival.

Evidence from transcriptomic, clinical, and experimental studies indicates that mitochondrial integrity is important for maintaining sleep homeostasis. It is becoming clear that mitochondrial dysfunction (due to genetic mutations, metabolic disease, or environmental toxins) plays a role in the development of insomnia, hypersomnia, abnormal circadian rhythms, and sleep fragmentation (74, 136).

The unified theory of sleep provides an intriguing perspective that supports the mitochondrial view. This theory proposes that sleep, a process that dampens the effects of cellular energy requirements and facilitates the integration of the host and symbiont, evolved with mitochondrial endosymbiosis (130). According to this view, sleep could be an ancient adaptation that enabled the eukaryotic cell and its mitochondrial symbiont to survive. Whereas rapid-eye movement (REM) sleep may be a balancing mechanism to preserve sensory awareness and behavioral responsiveness, non-REM sleep may be involved in mitochondrial recovery and genome integration.

To build mitochondrial-based hypotheses about sleep, we undertook a bioinformatics-based analysis to map 9 mitochondrial protein-coding genes related to sleep phenotypes and energy regulatory processes. Although the enrichment in OXPHOS and ATP synthesis pathways was expected, the analyses also revealed significant correlations with calcium signaling and synaptic plasticity, indicating a distinct function of mitochondria in sleep regulation. These pathways, which are frequently dysregulated in neurodegenerative and mental disorders with mitochondrial malfunction, are continuously hampered by sleep deprivation.

These correlations are also supported by experimental data. In both humans and animals, sleep deprivation causes altered mitochondrial shape, decreased ATP generation, increased reactive oxygen species (ROS), and compromised calcium buffering. Changes in sleep architecture and a higher incidence of insomnia are caused by mutations in genes such MCU1, which codes for the mitochondrial calcium uniporter (66, 77). These findings highlight the vicious cycle that exists within the neurobiological network between mitochondrial dysfunction and sleep problems, which exacerbate each other.

It is proposed that these could be effectively examined from the standpoint of mitochondrial activity, a concept that has been dubbed the “mitochondrial theory of sleep.” It is suggested that this theory’s model may open up new possibilities for creative therapeutic approaches. It is proposed that mitochondria-focused therapies could enhance resilience and sleep. Such approaches include dietary supplements like CoQ10 and PQQ, medication therapy like mitochondrial-specific antioxidants, and behavioral methods like exercise and circadian rhythm entrainment. Additionally, sleep vulnerabilities can be identified and precision medication can be guided by signs generated from mitochondria, such as metabolites, redox states, and mtDNA copy number.

Certain information holes still exist. The use of correlated data in the mitochondrial theory or model is one of its main drawbacks. Despite a substantial connection between sleep deprivation and mitochondrial dysfunction, there is currently little evidence of a direct relationship.

Future studies should use conditional knockouts, optogenetic manipulation of mitochondrial activity in pathways known to regulate sleep, and mitochondrial gene editing to experimentally demonstrate the causal relationship.

The role of mitochondria-associated membranes (MAMs) in sleep regulation is an exciting frontier that our framework highlights. MAMs are critical for calcium transfer, lipid metabolism, and ROS signaling, all processes implicated in sleep-wake regulation. Sleep deprivation-induced ER stress and MAM formation (84) suggest that ER-mitochondria communication may be a key mechanism linking cellular stress to sleep need (137). Future studies should investigate MAM dynamics across sleep-wake cycles and their potential as therapeutic targets.

Another critical need for research would be efforts to increase understanding of mitochondrial dynamics in sleep (42). Mitochondrial quality control is maintained by fission, fusion, and mitophagy, which may have stage-specific effects on REM and non-REM sleep. The mechanisms involved in these dynamics could be further explored by analyzing their dynamics during the sleep-wake cycle.

Gender, age, and factors such as pollution, food, and shift work are just some of the poorly understood factors that may influence mitochondrial function and hence sleep. The effects of these factors on the mitochondrial-sleep interface might explain individual differences in sleep need, adaptation, and vulnerability to sleep disorders.

There is a need for future empirical studies to advance the mitochondrial theory of sleep. As such, a multi-faceted approach should be adopted. This includes 1) Manipulation of mitochondrial genes (such as ATP6, ND1 or MCU1) *in vivo* in sleep-regulating areas of the brain; 2) Real-time monitoring of mitochondrial function during rapid eye movement (REM) and non-REM sleep transitions; 3) Integration of multi-omics data (such as transcriptomics, metabolomics, and epigenomics) to discover mitochondrial regulatory networks; 4) Clinical studies to test the efficacy of mitochondrial interventions in patients with primary sleep disorders or those with co-occurring sleep disorders; and 5) Biomarker development for sleep staging. Importantly, the aim of the mitochondrial sleep theory is not to replace existing theories and models but to complement them. It reinforces the neurophysiological, synaptic, and hormonal theories by offering a basic metabolic mechanism for their interaction. The theory proposes that sleep is a highly conserved cellular process that is rooted in the energy demands and constraints of mitochondrial life, not simply a neurophysiological process.

The identification of mitochondrial targets including MCU1, CPT1A, and antioxidant pathways offers promising avenues for future research. However, it is important to note that most of these targets have not been validated in clinical trials for sleep disorders. We propose that preclinical studies testing mitochondrial-targeted interventions in animal models of sleep deprivation and insomnia are a necessary next step before clinical translation (138).

The mitochondrial framework also offers a basis for understanding individual differences in sleep need and vulnerability. Factors such as mtDNA haplogroups, mitochondrial copy number, and genetic polymorphisms in mitochondrial regulators (e.g., MCU1, PPARα, CPT1A) may contribute to inter-individual variability in sleep homeostasis and response to sleep loss. This has implications for personalized sleep medicine and the identification of mitochondrial biomarkers.

A significant limitation of the current literature, and therefore of this review, is that much of the evidence remains correlative. While the associations between mitochondrial dysfunction and sleep disturbances are robust and reproducible across multiple models and species, direct causal evidence through, for example, conditional knockout of mitochondrial genes in sleep-regulating neurons or optogenetic manipulation of mitochondrial activity is still limited. We therefore emphasize that the mitochondrial theory of sleep should be viewed as a testable framework that generates specific predictions for future experimental studies.

The mitochondrial theory of sleep provides a robust and integrated framework for understanding sleep regulation. It is also part of an interdisciplinary study of translational neuroscience, molecular genetics, and evolution. A considerable amount of evidence suggests that the mitochondrion, a bacterial endosymbiont, has evolved into a sleep-wake master regulator. Gaining insight into its role in sleep offers a platform for determining the causes of sleep, understanding the origins of sleep disorders, and developing new therapies.

## 9 Conclusion

The mitochondrial theory of sleep proposes that a fundamental function of sleep is the preservation and restoration of mitochondrial function, and the primary causes of sleep dysfunction are mitochondrial signaling and metabolic dysfunctions. This theory expands on the evolutionary theory of sleep, such as the unified theory of sleep, which proposes that the evolution of sleep is related to the endosymbiotic inclusion of mitochondria into the early eukaryotic cell.

Our bioinformatics analysis supports this framework by identifying nine mitochondrial protein-coding genes associated with sleep via bioinformatics and literature review. Using bioinformatics techniques, such as gene ontology analysis and enrichment mapping of GO, Reactome, and KEGG databases, we demonstrate that these genes are enriched in pathways crucial for ATP synthesis, stress response, calcium homeostasis, and neurotransmitter production.

Beyond just being a good theory, the mitochondrial theory of sleep has potential benefits. Future research should explore whether modifying mitochondrial function through pharmacological, nutritional, or lifestyle interventions can improve sleep and stress resilience. Through our theory of sleep, we aim to provide an integrative paradigm that considers both molecular complexity and behavioral impacts by focusing on mitochondria.

In summary, We contend that sleep is not merely a result of neuronal networks or endocrine cycles, but rather a biologically conserved condition that is intrinsically linked to mitochondrial function and energetic homeostasis. Opportunities for more empirical study, therapeutic applications, and theoretical advancement based on a common molecular basis for many physiological and behavioral studies are presented by the mitochondrial theory of sleep.

Our bioinformatics method provides a means of methodically identifying functional and evolutionarily conserved mitochondrial genes or proteins associated with sleep, resulting in testable hypotheses and experimental work prioritization. By connecting molecular metabolism to brain function, the mitochondrial hypothesis of sleep offers a tenable, verifiable, and cohesive explanation of sleep.
