# Supplementary material for: The mitochondrial theory of sleep: an integrative framework for understanding sleep-wake regulation
Source: Front Psychiatry. 2026 Sep 7;17:1922556. doi: 10.3389/fpsyt.2026.1922556 (PMC13592155; doi:10.3389/fpsyt.2026.1922556)
Supplement: Supplementary file 1 [file Supplementaryfile1.docx]

**Supplementary Methods**

**Identification of Sleep-Associated Mitochondrial Genes**

The human mitochondrial genome contains 13 protein-coding genes encoding essential subunits of the oxidative phosphorylation (OXPHOS) system. To identify mitochondrial genes associated with sleep, a comprehensive literature-based search was performed using PubMed, Google Scholar, GeneCards, and OMIM (1–4). Search terms included combinations of sleep, sleep disorders, circadian rhythm, mitochondria, mitochondrial DNA, oxidative phosphorylation, and the names of individual mitochondrial genes (e.g., MT-ND1, MT-CO1, MT-CYB, and MT-ATP6). Only genes supported by published evidence demonstrating direct or indirect associations with sleep physiology, circadian regulation, or sleep-related disorders were retained. This process identified nine mitochondrial protein-coding genes: ND1, ND2, ND4, ND5, COX1, COX2, COX3, ATP6, and MT-CYB.

**Functional Enrichment Analysis**

Functional enrichment analysis was performed using the PESCADOR platform (5) to determine whether the identified sleep-associated mitochondrial genes were significantly overrepresented in specific biological pathways and functional categories. The nine sleep-associated genes were used as the input gene list, whereas the complete set of 13 human mitochondrial protein-coding genes served as the background reference. This background was selected to evaluate enrichment relative to the mitochondrial genome rather than the entire nuclear genome, thereby minimizing bias arising from mitochondrial gene composition.

The analysis included enrichment against three major annotation databases:

Gene Ontology (GO), including Biological Process (BP), Molecular Function (MF), and Cellular Component (CC) using gprofiler tool (6);

Kyoto Encyclopedia of Genes and Genomes (KEGG) pathways (7);

Reactome biological pathways (8).

Overrepresentation analysis was performed using the statistical framework implemented in PESCADOR. Statistical significance was calculated using the hypergeometric test, and multiple hypothesis testing was corrected using the Benjamini-Hochberg false discovery rate (FDR) procedure. Functional categories with an adjusted FDR < 0.05 were considered significantly enriched.

**Functional Network and Clustering Analysis**

To investigate functional relationships among the enriched pathways, PESCADOR-generated interaction networks were examined to identify genes participating in multiple biological processes (5). Functional hub genes were defined as genes connected to multiple significantly enriched pathways within the interaction network. Hierarchical clustering of enriched functional categories was also performed to identify biologically related modules and to visualize similarities among oxidative phosphorylation, respiratory electron transport, ATP synthesis, and other mitochondrial processes.

**Disease Association Analysis**

Disease enrichment analysis was carried out using disease annotations integrated within the DisGeNet database (9). Significant associations between mitochondrial genes and human diseases were identified based on curated disease databases. Enriched disease categories were evaluated using the same statistical framework described above, with Benjamini-Hochberg correction applied to account for multiple comparisons.

**References**

1.	Falagas ME, Pitsouni EI, Malietzis GA, Pappas G. Comparison of PubMed, Scopus, Web of Science, and Google Scholar: strengths and weaknesses. *FASEB J* (2008) 22:338–342. doi: https://doi.org/10.1096/fj.07-9492LSF

2.	Jin Q, Leaman R, Lu Z. PubMed and beyond: biomedical literature search in the age of artificial intelligence. *eBioMedicine* (2024) 100:104988. doi: 10.1016/j.ebiom.2024.104988

3.	Stelzer G, Rosen N, Plaschkes I, Zimmerman S, Twik M, Fishilevich S, Stein TI, Nudel R, Lieder I, Mazor Y, et al. The GeneCards Suite: From Gene Data Mining to Disease Genome Sequence Analyses. *Curr Protoc Bioinforma* (2016) 54:1.30.1-1.30.33. doi: https://doi.org/10.1002/cpbi.5

4.	Hamosh A, Scott AF, Amberger JS, Bocchini CA, McKusick VA. Online Mendelian Inheritance in Man (OMIM), a knowledgebase of human genes and genetic disorders. *Nucleic Acids Res* (2005) 33:D514–D517. doi: 10.1093/nar/gki033

5.	Barbosa-Silva A, Fontaine J-F, Donnard ER, Stussi F, Ortega JM, Andrade-Navarro MA. PESCADOR, a web-based tool to assist text-mining of biointeractions extracted from PubMed queries. *BMC Bioinformatics* (2011) 12:435. doi: 10.1186/1471-2105-12-435

6.	Reimand J, Kull M, Peterson H, Hansen J, Vilo J. g:Profiler—a web-based toolset for functional profiling of gene lists from large-scale experiments. *Nucleic Acids Res* (2007) 35:W193–W200. doi: 10.1093/nar/gkm226

7.	Kanehisa M, Goto S. KEGG: Kyoto Encyclopedia of Genes and Genomes. *Nucleic Acids Res* (2000) 28:27–30. doi: 10.1093/nar/28.1.27

8.	Milacic M, Beavers D, Conley P, Gong C, Gillespie M, Griss J, Haw R, Jassal B, Matthews L, May B, et al. The Reactome Pathway Knowledgebase 2024. *Nucleic Acids Res* (2024) 52:D672–D678. doi: 10.1093/nar/gkad1025

9.	Piñero J, Bravo À, Queralt-Rosinach N, Gutiérrez-Sacristán A, Deu-Pons J, Centeno E, García-García J, Sanz F, Furlong LI. DisGeNET: a comprehensive platform integrating information on human disease-associated genes and variants. *Nucleic Acids Res* (2017) 45:D833–D839. doi: 10.1093/nar/gkw943
